# Assessment of the treating physicians’ first-hand experience with handling and satisfaction of ofatumumab therapy: findings from the PERITIA survey conducted in Europe

**DOI:** 10.1186/s12883-023-03190-x

**Published:** 2023-04-10

**Authors:** Daniela Rau, Sara Eichau, Giovanna Borriello, João Cerqueira, Carola Wagner

**Affiliations:** 1 NeuroPoint GmbH, 89073 Ulm, Germany; 2 Hospital Universitario Virgen de La Macareona of Sevilla, Seville, Spain; 3 MS Center, Neurology Unit, Fatebenefratelli San Pietro Hospital, Rome, Italy; 4 2CA Centro Clínico Académico, Braga, Portugal; 5 Novartis Pharma GmbH, Nuremberg, Germany and working on behalf of Novartis Pharma Vertriebs GmbH, Nuremberg, Germany

**Keywords:** Ofatumumab, Multiple sclerosis, Pre-filled syringe, Self-medication, Convenience, Treatment satisfaction

## Abstract

**Background:**

Real-world evidence on experience and satisfaction of ofatumumab as a treatment option for relapsing multiple sclerosis (RMS) is limited.

**Objective:**

To present cumulative responses from a questionnaire related to first-hand experience of treating physicians on handling and convenience of ofatumumab therapy along with concerns related to COVID-19.

**Methods:**

PERITIA was a multicentre survey conducted to collect responses from the ASCLEPIOS I/II trial investigators from Europe via an online questionnaire.

**Results:**

Forty-six physicians (Germany, *n* = 14; Spain, *n* = 12; Portugal, *n* = 10; Italy, *n* = 10) completed the survey. Overall, 43% of the physicians considered the benefit-risk ratio of ofatumumab as very good. Over 93% were in favour of ofatumumab self-administration at home and the majority (83%) believed it to be completely true that self-administration of ofatumumab eases the burden for patients in terms of time. All investigators would like to potentially use anti-CD20 therapy as a long-term strategy. Even during the COVID-19 pandemic, physicians were in favour of a self-administration of MS therapy at home over other anti-CD20 therapy infusions.

**Conclusion:**

European neurologists who were part of this survey considered the benefit-risk-ratio of ofatumumab as favourable and the monthly self-administered subcutaneous injections offering convenience for patients in the clinical practice.

**Supplementary Information:**

The online version contains supplementary material available at 10.1186/s12883-023-03190-x.

## Introduction

Multiple sclerosis (MS) is a chronic inflammatory disease of the central nervous system characterised by myelin destruction and axonal damage in the brain, optic nerves and spinal cord [1]. MS affects approximately 2.8 million people worldwide as per 2020 statistics [2].

B cells activate T cells in lymph nodes and play a major role in the pathophysiology of MS [3]. Ofatumumab is a fully human anti-cluster of differentiate 20 (CD20) monoclonal antibody which binds to the CD20 molecule on the B-cell surface, and induces potent B-cell lysis and depletion [4]. In a preclinical study, relatively low-dose subcutaneous (SC) treatment with ofatumumab (20 μg/mouse) effectively depleted B cells in the blood, lymph nodes and spleen within 72 h, and B-cell repletion was more rapid after treatment cessation compared with a 7.5 times higher intravenous therapy (150 μg/mouse) [5]. The selective mechanism of action and SC administration of ofatumumab (which allows high molecular proteins reach the lymphatic system, primary site of interaction of B and T cells, in a targeted manner) [6, 7] allow precise delivery to the lymph nodes while relatively sparing B cells in the spleen which may help maintain protective immunity [8–10]. Ofatumumab differs from other anti-CD20 therapies as it allows faster repletion of B cells, offering more flexibility in MS management [11, 12].

Ofatumumab (KESIMPTA^®^) 20 mg once-monthly SC injection has been approved in the United States (US) in August 2020 [13], and in several other countries for the treatment of RMS in adults. In the European Union (EU), ofatumumab was approved in March 2021 [14] for the treatment of adult patients with RMS with active disease defined by clinical or imaging features. The approval of ofatumumab was based on the results from the pivotal Phase 3 ASCLEPIOS I and II trials [15]. Ofatumumab is the first approved targeted B-cell therapy that can be self-administered at home as a once-monthly SC injection via a pre-filled syringe or the Sensoready^®^ autoinjector pen [15–17]. The initial dose is given under healthcare provider (HCP) supervision [13, 14].

Real-world data on experience and satisfaction as well as implementation of ofatumumab as a therapy for RMS are limited and are not part of the pivotal ASCLEPIOS I and II trials [15]. As ofatumumab has been recently approved, treating physicians’ first-hand experience on ofatumumab therapy from the pivotal trials could be helpful while treating RMS patients with ofatumumab in clinical practice. Furthermore, there are uncertainties among patients and physicians regarding the impact of MS, especially with the use of immuno-modulatory/suppressive MS therapies, on the risk for COVID-19 infection and complications [18, 19].

In this survey, investigators were asked to share their personal experience and evaluation of ofatumumab therapy, such as handling, experience, route of administration, premedication and first-dose administration along with concerns related to COVID-19 via an online questionnaire from German, Spanish, Portuguese and Italian ASCLEPIOS Phase 3 study investigators.

## Methods

### Survey design and participants

PERITIA was a multicentre survey conducted in ASCLEPIOS I/II trial centres from four European countries: Germany, Spain, Portugal and Italy. Investigators were selected for the survey if they had recruited patients in the ASCLEPIOS I/II trials (NCT02792218 and NCT02792231). If the centres had no patients randomised to receive ofatumumab, they were selected only if they had participated in the ALITHIOS trial (NCT03650114), an extension Phase 3 trial evaluating the long-term safety, tolerability and effectiveness of ofatumumab in patients with RMS who had participated in Novartis ofatumumab clinical MS study.

Data were collected through the responses of eligible investigators to the online questionnaire in the form of a single answer, multiple answers or free text. The survey link was sent to the eligible investigators via email. German investigators received and responded to the survey between July and November 2020, and the remaining investigators, between February and April 2021. The data were collected from German investigators in German and from the investigators from Italy, Spain and Portugal in English.

### Statistical analysis

Both quantitative and qualitative data collection and analysis were part of this survey. Survey results are summarised descriptively, and categorical variables are summarised using frequencies and percentages.

### Questionnaires

Respondents were interviewed on 47 questions on different parameters, mainly investigators’ experience with and real-world implementation of ofatumumab as a therapy for RMS in real life. Impact of the COVID-19 pandemic on e.g. patient’s behaviour or change in treatment was also included in the questionnaire. The questions were single choice, multiple choice, multiple open text and array type. The survey questionnaire is presented in the Supplementary material.

## Results

### Survey participants

In total, all 46 physicians who were sent the survey questions, completed the survey (Germany, *n* = 14; Spain, *n* = 12; Portugal, *n* = 10; Italy, *n* = 10). The study sites were either at a practice/community health centre (24%, 11/46) or at a hospital/outpatient clinic (76%, 35/46). In total, 41% (19/46) of the physicians had > 20 years, 46% (21/46) had > 10–20 years and 13% (6/46) had < 10 years, of experience in treating MS patients. As there were no major differences observed between cumulative responses from investigators participating from the four countries, the overall data are presented.

### Perceived safety

The benefit-risk ratio of ofatumumab was assessed as either very good (43%, 20/46) or good (57%, 26/46) by the investigators. None of the investigators gave neutral, poor or very poor as an answer. Most of the treating physicians (87%, 40/46) believed that the precise low dose of ofatumumab (20 mg) and the SC administration of ofatumumab, which promotes specific targeting of the lymph nodes, are perceived advantages over other anti-CD20 therapies. Across all four countries, 91% (42/46) of the treating physicians responded positively when asked if the shorter B-cell repletion time after discontinuing ofatumumab therapy [12] and, consequently, higher flexibility in treatment are perceived advantages over other anti-CD20 therapies. Overall, 80% (37/46) of the treating physicians responded that this would impact on their choice of therapy.

### Route of administration (includes self-administration)

**Fig. 1:**
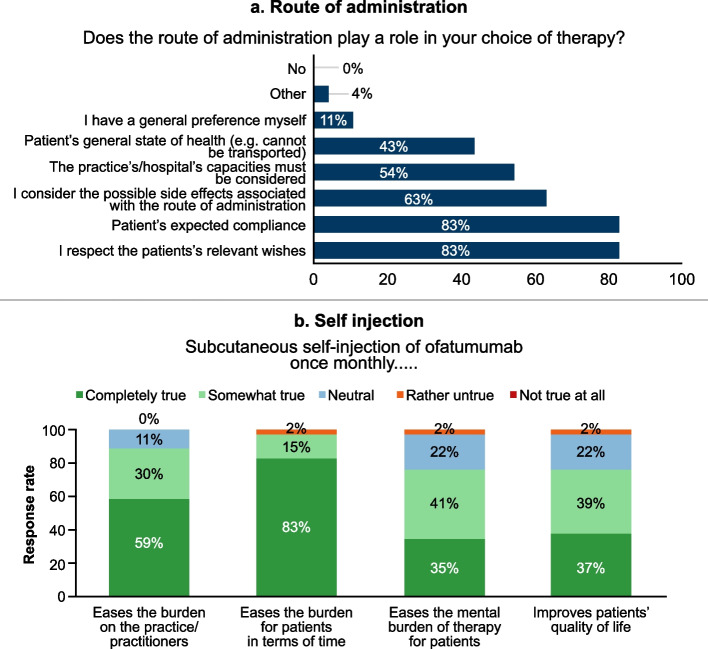
Responses related to questions on ofatumumab route of administration

While determining the treatment for patients, the appropriate route of administration played a role as follows: 83% (38/46) of the physicians agreed with the statements that they respect patients’ relevant wishes and consider the patient’s expected compliance, 63% (29/46) responded that they consider possible side effects associated with route of administration and 54% (25/46) believed that capacity at the treating site must be considered (Fig. 1a). Almost all of the physicians (98%, 45/46) believed that the SC route of ofatumumab administration is an advantage, and 83% (38/46) agreed that the SC route of administration will motivate them to use ofatumumab more frequently.

Even though the treating physicians had the ofatumumab pre-filled syringes used in the ASCLEPIOS trials in mind, the results regarding improvement of patients’ quality of life using the SC self-administration were positive (Fig. 1b). To the statement that ‘SC self-administration of ofatumumab eases the burden for patients in terms of time’, most of the physicians (83%, 38/46) believed it to be completely true, 15% (7/46) believed it to be somewhat true and 2% (1/46) stated it as rather untrue. To the statement that ‘SC self-administration of ofatumumab eases the burden on the practice/practitioners’, 59% (27/46) believed it to be completely true, 30% (14/46) believed it to be somewhat true and 11% (5/46) were neutral. To the statement that ‘SC self-administration of ofatumumab eases the mental burden of therapy for patients’, 35% (16/46) believed it to be completely true, 41% (19/46) believed it to be somewhat true, 22% (10/46) were neutral and 2% (1/46) stated it as rather untrue. To the statement that ‘SC self-administration of ofatumumab improves patients’ quality of life’, 37% (17/46) believed it to be completely true, 39% (18/46) believed it to be somewhat true, 22% (10/46) were neutral and 2% (1/46) stated it as rather untrue.

Most of the physicians (93%, 43/46) were in favour of self-administration of ofatumumab at home after marketing authorisation of ofatumumab is granted. Despite having experience with the pre-filled syringe in the ASCLEPIOS trials and not the Sensoready^®^ autoinjector pen, 30% (13/43) preferred self-administration of ofatumumab at home after the first injection, 12% (5/43) after the second injection, 37% (16/43) after the third injection and 14% (7/43) after four or more injections. Two of the 43 investigators (5%) did not specify the time point of self-administration at home.

### Monitoring, premedication and therapy algorithm

Based on the knowledge at the time of the survey, 40% (18/46) (includes responses ‘yes, it will be my preferred therapeutic option’, ‘yes, frequently’) of the treating physicians would use ofatumumab in the therapeutic algorithm for treatment-naïve patients, 87% (40/46) for first escalation therapy and 68% (31/46) for second escalation therapy.

**Fig. 2:**
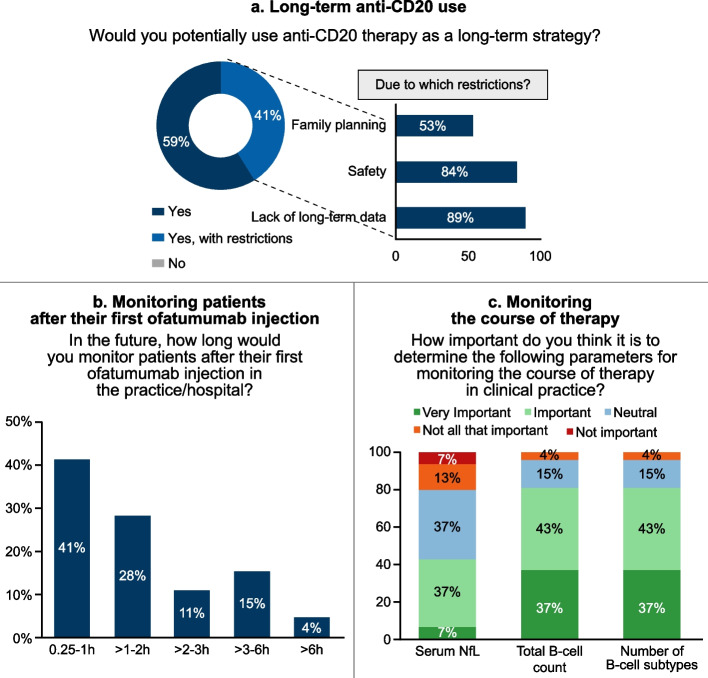
Responses related to questions on monitoring, premedication and therapy algorithm

All investigators confirmed that they would like to potentially use anti-CD20 therapy as a long-term strategy. Among them, 41% (19/46) investigators would use with one or more of the following restrictions: lack of long-term data (89%, *n* = 17), safety (84%, *n* = 16) and family planning (53%, *n* = 10) (Fig. 2a).

Overall, 41% (19/46) of the physicians responded that they would monitor patients for 0.25–1 h after the first ofatumumab injection in the hospital/practice, whereas 28% (13/46) would monitor for > 1–2 h and 30% (14/46) for > 2 h (Fig. 2b).

During the course of therapy in clinical practice, treating physicians considered determining total B-cell count and number of B-cell types for monitoring the course of therapy in clinical practice as very important (37% [17/46] each) or important (43% [20/46] each), while serum neurofilament light (NfL) as very important by 7% (3/46) and important by 37% (17/46) (Fig. 2c).

Most of the physicians would prefer to use antihistamines (89%, 42/46), steroids (94%, 44/46) or paracetamol (85%, 40/46) as premedication before administering the first ofatumumab injection in clinical routine; 11%–40% of the physicians preferred premedication before the second injection and 4%–28% preferred premedication before the third injection.

### COVID-19 pandemic

**Fig. 3:**
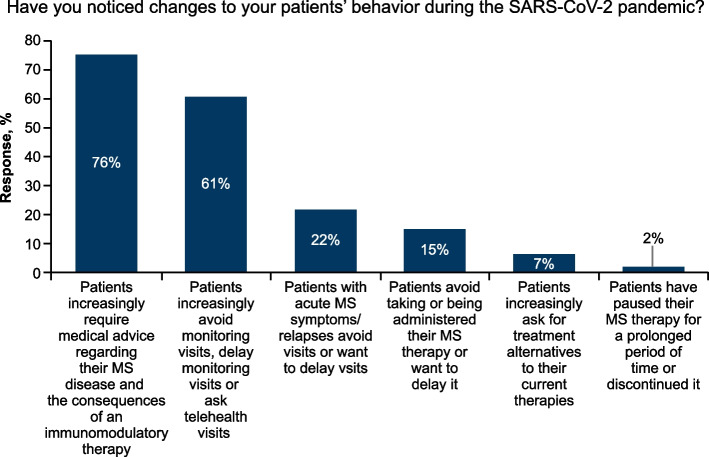
Responses related to a question on COVID-19 pandemic in relation to changes in patients’ behaviour

During the COVID-19 pandemic, 76% (35/46) of the physicians noticed that an increasing number of patients required medical advice about their MS and the consequences of an immunomodulatory therapy; 61% (28/46) noticed that patients increasingly avoided monitoring visits, delayed monitoring visits at the practice/hospital or asked for telehealth visits and 22% (10/46) noticed that patients with acute MS symptoms/relapses avoided visits or wanted to delay visits (Fig. 3).

During the COVID-19 pandemic, some of the physicians made changes to the treatment for MS patients. Few physicians responded that in the majority of the cases, they postponed patient’s monitoring visits (4%; 2/46), postponed the infusion appointments (2%; 1/46) and initiated alternative therapies that patients could take at home (2%; 1/46).

During the COVID-19 pandemic, 98% (45/46) of physicians had no concerns about the use of interferon-β and glatiramer acetate; 61% (28/46) had no concerns about the use of dimethyl fumarate (DMF), teriflunomide, fingolimod and siponimod; and 17% (8/46) had no concerns about the use of cladribine, natalizumab, alemtuzumab and ocrelizumab.

During the COVID-19 pandemic, 50% of the treating physicians were of the opinion that a longer stay in the hospital/practice, such as during a DMT infusion or a clinical study visit, constitutes a risk. As high as 89% (41/46) of the treating physicians saw self-administering MS therapy or taking it at home as an advantage over MS therapies administered as infusions.

## Discussion

This survey was designed to better understand the experience and satisfaction of ofatumumab therapy from ASCLEPIOS study investigators’ perspective because there are limited data available in the clinical practice. As ofatumumab has been recently approved, the findings reported in the current survey provide further cumulative insights from treating physicians about their first-hand experience on the handling and satisfaction of ofatumumab therapy while managing people living with MS. In addition, these survey results provide insights on the general concerns, preferences and changes in patients’ behaviour during the COVID-19 pandemic.

All the investigators assessed the benefit-risk ratio of ofatumumab as either very good (43%) or good (57%) and none of them responded it to be neutral, poor or very poor. This cumulative response supports the previous findings from the Phase 3 ASCLEPIOS I and II trials, where ofatumumab demonstrated a significant relative reduction in the annualised relapse rate, significant suppression of both Gd + T1 lesions and number of new or enlarging T2 lesions, significant reduction in confirmed disability worsening and a favourable safety profile versus teriflunomide [15].

Monitoring after the first-dose administration was mandatory in ASCLEPIOS I and II trials [15]. In this survey, approximately 40% of the treating physicians intend to monitor patients for < 1 h after the first ofatumumab administration, whereas in ASCLEPIOS I and II trials, patients were required to remain at the site under observation for a minimum of 5 h following the first dose [15]. There is no formal obligation for a safety follow-up after the first dose administration of ofatumumab in the US or EU labels [13, 14].

In ASCLEPIOS I and II trials [15], at the discretion of the investigator, premedication with acetaminophen and/or antihistamines (or equivalent) was recommended, and for the first injection only, the addition of steroids (intravenous methylprednisolone 100 mg or equivalent) was recommended. Similar to the findings of the ASCLEPIOS I and II trials [15], the physicians’ preference of premedication usage declined after the first injection in the current survey results. Thus, the results are in line with approved labels where injection-related reactions can be managed with symptomatic treatment, if they occur, and only a limited benefit of premedication was observed with corticosteroids, antihistamines or acetaminophen (US label [13]) and with steroids (EU label [14]). In addition, the EU label states that the use of premedication is not required [14].

Per the treating physicians, patients’ preference or expected compliance in terms of route of administration is largely taken into account when making treatment choices. Most treating physicians were in favour of the SC route of administration and self-administration.

In this survey, notably, almost 80% (34/43) of the study investigators would support self-administration at home after the 1st-3rd injection in clinical routine. A recent article by Filippi M et al. mentioned that use of oral and self-administered DMTs could lead to reduction in costs for health care systems mainly in terms of hospital occupation, and in the risk of infection; additionally, it could attenuate the difficulties in access to care caused by the COVID-19 pandemic [20]. Per the ofatumumab label, the first injection of ofatumumab should be performed under the guidance of an appropriately trained HCP. Thereafter, ofatumumab is intended for patient self-administration [13, 14].

The ASCLEPIOS I and II trials comprised of both treatment-naïve and previously treated patients [15]. In the subgroup analysis of newly diagnosed and treatment-naïve patients, ofatumumab showed superior efficacy versus teriflunomide, which was consistent with the overall ASCLEPIOS I and II study population [15, 21]. In this survey, we found that 40% of the treating physicians would favour the use of ofatumumab for treatment-naïve patients; 87% prefer to use ofatumumab as first escalation therapy and 68% prefer to use it as second escalation therapy. A recently published article determined that an early and unrestricted access to high-efficacy (HE) DMTs including ofatumumab for people living with MS in Europe, showed a positive benefit-risk profile and improved outcomes indicating that the early use is the best strategy to delay the progression of MS [20]. HEDMTs (including alemtuzumab, natalizumab, cladribine, fingolimod, ocrelizumab, and ofatumumab) have demonstrated improved efficacy versus interferons or teriflunomide in reducing relapse rates, MRI lesions, brain volume loss, and/or delaying disease progression across several clinical programs [15, 22–27].

In the Phase 3 ASCLEPIOS trials [9], ofatumumab was administered in a pre-filled syringe; however, ofatumumab is approved in Europe [14] as a pre-filled syringe and an autoinjector pen. Injection tolerability and patient satisfaction due to convenience of administration is improved with autoinjectors versus manual injection, thereby increasing adherence [28, 29]. In a recent survey, both patients and nurses preferred the Sensoready^®^ autoinjector pen for ofatumumab 20 mg SC administration over other autoinjectors for their current treatments. This was mainly due to the ease of use when self-injecting with the pen and the ability of the patient to use it independently [16]. The findings of the present survey showed that the handling and experience of ofatumumab with a pre-filled syringe makes it the preferred dosage form, and it is anticipated that the first-hand experience could have surpassed these findings with the pre-filled Sensoready^®^ autoinjector pen dosage form. Of note, this survey was initiated and completed by most of the European HCPs prior to the approval of ofatumumab in Europe.

The current findings from this survey are complementary to the ASCLEPIOS I and II trials findings [15]; however, this survey was not part of the trials and was independent of these studies.

During the COVID-19 pandemic, the treating physicians favoured ofatumumab SC self-administration at home over other anti-CD20 therapies administered as infusions. Most (95%) of the treating physicians preferred alternative therapies that the patient could take/use at home. In the ongoing ALITHIOS trial, there was no increased risk of severe or serious COVID-19 in people with MS versus general population [30].

Limitations of this survey include that only investigators from ASCLEPIOS I and II trials were involved who gained the ofatumumab treatment experience during the conduct of the ASCLEPIOS clinical trials and not during clinical practice. Further limitation is the fact that these findings were experiences by the investigators during the duration of the ASCLEPIOS I and II trials with a follow-up of up to 5 years, so this survey is only an early feeling to re-evaluate in the future. Furthermore, a detailed inquiry of the risk and safety assessment for ofatumumab was not part of this survey, and investigators from a limited number of countries were involved. Also, the survey was conducted in European countries and Europe has widely accessible healthcare when compared with other developed countries, and thus present results do not necessarily apply elsewhere.

In summary, after gaining experience with ofatumumab within clinical trials, European neurologists who were part of this survey considered ofatumumab to be a very efficient and safe treatment option. They agreed that the self-administered once-monthly SC administration injection offers convenience for patients and facilitates processes at high-occupancy clinics and office-based practices. As a future perspective, this survey could be conducted in a real-word setting to collect data related to physicians’ experience on the treatment-related clinical parameters and could be extended to all the MS healthcare professionals in the clinical practice to gain further knowledge.

## Supplementary Information

**Additional file 1. **Supplemental material.

## Data Availability

The data that support the findings of this survey are available from [Novartis Pharma GmbH and Novartis Pharma AG] but restrictions apply to the availability of these data, which were used under license for the current survey, and so are not publicly available. Data are however available from Carola Wagner (carola.wagner@novartis.com) upon reasonable request and with permission of [Novartis Pharma GmbH and Novartis Pharma AG].
