# Supplementary material for: Assessment of the treating physicians’ first-hand experience with handling and satisfaction of ofatumumab therapy: findings from the PERITIA survey conducted in Europe
Source: BMC Neurol. 2023 Apr 10;23:147. doi: 10.1186/s12883-023-03190-x (PMC10084644; doi:10.1186/s12883-023-03190-x)
Supplement: Supplementary file 1 — Additional file 1. Supplemental material. [file 12883_2023_3190_MOESM1_ESM.docx]

**Supplemental material**

Page 1

| Q01 | **Which type of institution are you a part of?** | | | | |  |
| --- | --- | --- | --- | --- | --- | --- |
|  | Surgery / Community Health Centre | | | | Q01-1 |  |
|  | Hospital / Outpatient Clinic | | | | Q01-2 |  |
|  | Rehab Facility | | | | Q01-3 |  |
|  | | | | | | |
| Condition | | N/A |  |  | | |
| Question type | | Single Choice |  |  | | |
| Validation | | No |  |  | | |

| Q02 | **You have stated “Hospital / outpatient clinic”. Please specify your response.**  Multiple responses possible | | | | |  |
| --- | --- | --- | --- | --- | --- | --- |
|  | University Hospital | | | | Q02-1 |  |
|  | Acute Hospital | | | | Q02-2 |  |
|  | University’s Outpatient Clinic | | | | Q02-3 |  |
|  | Outpatient Clinic as per Sec. 116-B SGB [German Social Code] | | | | Q02-4 |  |
|  | Other | | | | Q02-5 |  |
|  | |  |  |  | | |
| Condition | | IF Q01=2 |  |  | | |
| Question type | | Multiple Choice |  |  | | |
| Validation | | No |  |  | | |

| Q03 | **Is your site a Neurology Centre focussing on MS?**  Multiple responses possible | | | | |  |
| --- | --- | --- | --- | --- | --- | --- |
|  | DMSG certificate for MS Centres | | | | Q03-1 |  |
|  | DMSG certificate for Centres focussing on MS | | | | Q03-2 |  |
|  | DMSG certificate for MS Rehab Facilities | | | | Q03-3 |  |
|  | No certification (exclusive answer) | | | | Q03-4 |  |
|  | Other certificates (Open text) | | | | Q03-5 |  |
|  | |  |  |  | | |
| Condition | | N/A |  |  | | |
| Question type | | Multiple Choice |  |  | | |
| Validation | | No |  |  | | |

| Q04 | **How many years of experience treating MS patients do you have?** | | | | |  |
| --- | --- | --- | --- | --- | --- | --- |
|  |  | | | | Q04-1 |  |
|  | |  |  |  | | |
| Condition | | N/A |  |  | | |
| Question type | | Numeric Entry |  |  | | |
| Validation | | No |  |  | | |

| Q05 | **What was your role in the ASCLEPIOS study?** | | | | |
| --- | --- | --- | --- | --- | --- |
|  | Principal Investigator | | | | Q05-1 |
|  | Sub-Investigator | | | | Q05-2 |
|  | Co-Investigator | | | | Q05-3 |
|  | |  |  |  | |
| Condition | | N/A |  |  | |
| Question type | | Single Choice |  |  | |
| Validation | | No |  |  | |

| Q06 | **How many patients did you treat as part of the ASCLEPIOS study?** | | | | |  |
| --- | --- | --- | --- | --- | --- | --- |
|  | 1 | | | | Q06-1 |  |
|  | 2-5 | | | | Q06-2 |  |
|  | >5 | | | | Q06-3 |  |
|  | |  |  |  | | |
| Condition | | N/A |  |  | | |
| Question type | | Single Choice |  |  | | |
| Validation | | No |  |  | | |

| Q07 | **Is your study site also taking part in the ALITHIOS study (COMB157G2399)?** | | | | |  |
| --- | --- | --- | --- | --- | --- | --- |
|  | Yes | | | | Q07-1 |  |
|  | No | | | | Q07-2 |  |
|  | |  |  |  | | |
| Condition | | N/A |  |  | | |
| Question type | | Single Choice |  |  | | |
| Validation | | No |  |  | | |

Page 2

|  | **Please respond to the following questions based on your experience with ofatumumab to date.** |
| --- | --- |

| Q08 | **Ofatumumab is the first monoclonal antibody in MS therapy that does not need to be given as an infusion.**  **Do you think the subcutaneous route of administration is an advantage?** | | | | |
| --- | --- | --- | --- | --- | --- |
|  | Yes | | | | Q08-1 |
|  | No | | | | Q08-2 |
|  | |  |  |  | |
| Condition | | N/A |  |  | |
| Question type | | Single Choice |  |  | |
| Validation | | No |  |  | |

| Q09 | **Would subcutaneous administration be a motivation for you to use ofatumumab more frequently?** | | | | |  |
| --- | --- | --- | --- | --- | --- | --- |
|  | Yes | | | | Q09-1 |  |
|  | No | | | | Q09-2 |  |
|  | |  |  |  | | |
| Condition | | N/A |  |  | | |
| Question type | | Single Choice |  |  | | |
| Validation | | No |  |  | | |

| Q10 | **Please evaluate the following statements.**  **Subcutaneous self-injection of ofatumumab once monthly …** | | | | | | | | |  |
| --- | --- | --- | --- | --- | --- | --- | --- | --- | --- | --- |
|  | **Completely true** | **Somewhat true** | | **Neutral** | **Rather untrue** | | **Not true at all** | | |  |
|  | … eases the burden on the surgery/practitioners | | | | | | | | Q10-1 |  |
|  | … eases the burden for patients in terms of time | | | | | | | | Q10-2 |  |
|  | … eases the mental burden of therapy for patients | | | | | | | | Q10-3 |  |
|  | … improves patients’ quality of life | | | | | | | | Q10-4 |  |
|  | | |  | | |  | |  | | |
| Condition | | | N/A | | |  | |  | | |
| Question type | | | Array | | |  | |  | | |
| Validation | | | No | | |  | |  | | |

| Q11 | **How satisfied were you overall with the treatment with ofatumumab?** | | | | |
| --- | --- | --- | --- | --- | --- |
|  | Very satisfied | | | | Q11-1 |
|  | Satisfied | | | | Q11-2 |
|  | Neutral | | | | Q11-3 |
|  | Not very satisfied | | | | Q11-4 |
|  | Not satisfied | | | | Q11-5 |
|  | |  |  |  | |
| Condition | | N/A |  |  | |
| Question type | | Single Choice |  |  | |
| Validation | | No |  |  | |

| Q12 | **Does ofatumumab constitute a therapy suited to daily life in your opinion?** | | | | |  |
| --- | --- | --- | --- | --- | --- | --- |
|  | Yes | | | | Q12-1 |  |
|  | No | | | | Q12-2 |  |
|  | |  |  |  | | |
| Condition | | N/A |  |  | | |
| Question type | | Single Choice |  |  | | |
| Validation | | No |  |  | | |

| Q13 | **How would you assess the benefit-risk ratio of ofatumumab?** | | | | |  |
| --- | --- | --- | --- | --- | --- | --- |
|  | Very good | | | | Q13-1 |  |
|  | Good | | | | Q13-2 |  |
|  | Neutral | | | | Q13-3 |  |
|  | Poor | | | | Q13-4 |  |
|  | Very poor | | | | Q13-5 |  |
|  | |  |  |  | | |
| Condition | | N/A |  |  | | |
| Question type | | Single Choice |  |  | | |
| Validation | | No |  |  | | |

| Q14 | **The precise dosage of ofatumumab has been determined in a dose-effect study, and is 20mg. Due to the subcutaneous route of administration, ofatumumab allows the lymph nodes to be targeted at a lower dose.**  **Do you think the lower dose of ofatumumab is an advantage over other anti-CD20 therapies?** | | | | |  |
| --- | --- | --- | --- | --- | --- | --- |
|  | Yes | | | | Q14-1 |  |
|  | No | | | | Q14-2 |  |
|  | |  |  |  | | |
| Condition | | N/A |  |  | | |
| Question type | | Single Choice |  |  | | |
| Validation | | No |  |  | | |

| Q15 | **What are, in your opinion, the effects of directly addressing lymphatic tissues by subcutaneous administration of an immunomodulatory medicine?**  Multiple responses possible | | | | |  |
| --- | --- | --- | --- | --- | --- | --- |
|  | Faster effect of the immunomodulatory medicine possible | | | | Q15-1 |  |
|  | Fewer side effects expected | | | | Q15-2 |  |
|  | Has no clinical relevance (Exclusive) | | | | Q15-3 |  |
|  | Other (Open text) | | | | Q15-4 |  |
|  | |  |  |  | | |
| Condition | | N/A |  |  | | |
| Question type | | Multiple Choice |  |  | | |
| Validation | | No |  |  | | |

| Q16 | **Does the route of administration of a medicine play a role in your choice of therapy?**  Multiple responses possible | | | | |  |
| --- | --- | --- | --- | --- | --- | --- |
|  | I respect the patient’s relevant wishes | | | | Q16-1 |  |
|  | I have a general preference myself | | | | Q16-2 |  |
|  | The surgery’s/hospital’s capacities must be considered | | | | Q16-3 |  |
|  | I consider the possible side effects associated with the route of administration | | | | Q16-4 |  |
|  | Patient’s expected compliance | | | | Q16-5 |  |
|  | Patient’s general state of health (e.g. cannot be transported) | | | | Q16-6 |  |
|  | No (Exclusive) | | | | Q16-7 |  |
|  | Other (Open text) | | | | Q16-8 |  |
|  | |  |  |  | | |
| Condition | | N/A |  |  | | |
| Question type | | Multiple Choice |  |  | | |
| Validation | | No |  |  | | |

| Q17 | **You have stated “I have a general preference myself.”**  **Please specify your response.** | | | | |  |
| --- | --- | --- | --- | --- | --- | --- |
|  | Oral | | | | Q17-1 |  |
|  | Injection | | | | Q17-2 |  |
|  | Infusion | | | | Q17-3 |  |
|  | |  |  |  | | |
| Condition | | IF Q16=2 |  |  | | |
| Question type | | Single Choice |  |  | | |
| Validation | | No |  |  | | |

| Q18 | **Do you see the shorter B-cell repletion time after discontinuing ofatumumab therapy and the consequently higher flexibility in treatment as an advantage over other anti-CD20 therapies?**  **See publication here.**  („hier“ embedded with link: <https://onlinelibrary.ectrims-congress.eu/ectrims/2017/ACTRIMS-ECTRIMS2017/199644/david.leppert.comparison.of.the.b-cell.recovery.time.following.discontinuation.html>) | | | | |  |
| --- | --- | --- | --- | --- | --- | --- |
|  | Yes | | | | Q18-1 |  |
|  | No | | | | Q18-2 |  |
|  | |  |  |  | | |
| Condition | | N/A |  |  | | |
| Question type | | Single Choice |  |  | | |
| Validation | | No |  |  | | |

| Q19 | **Does the shorter B-cell repletion time after discontinuing ofatumumab therapy have an impact on your choice of therapy?** | | | | |  |
| --- | --- | --- | --- | --- | --- | --- |
|  | Yes | | | | Q19-1 |  |
|  | No | | | | Q19-2 |  |
|  | |  |  |  | | |
| Condition | | N/A |  |  | | |
| Question type | | Single Choice |  |  | | |
| Validation | | No |  |  | | |

| Q20 | **Do you think treatment with the highly effective medicine ofatumumab makes sense in patients having just experienced the onset of their MS ?** | | | | |  |
| --- | --- | --- | --- | --- | --- | --- |
|  | Yes, for all patients | | | | Q20-1 |  |
|  | Yes, for patients with high disease activity | | | | Q20-2 |  |
|  | No | | | | Q20-3 |  |
|  | |  |  |  | | |
| Condition | | N/A |  |  | | |
| Question type | | Single Choice |  |  | | |
| Validation | | No |  |  | | |

| Q21 | **Based on the current state of knowledge, would you use ofatumumab in the therapeutic algorithm?** | | | | | | | | |  |
| --- | --- | --- | --- | --- | --- | --- | --- | --- | --- | --- |
| Yes, it will be my preferred therapeutic option | | Yes, frequently | | Yes, but only in individual patients | Not at first, only after experience with another patient group | | No | | |  |
|  | In naïve patients | | | | | | | | Q21-1 |  |
|  | As first escalation therapy | | | | | | | | Q21-2 |  |
|  | As second escalation therapy | | | | | | | | Q21-3 |  |
|  | | |  | | |  | |  | | |
| Condition | | | N/A | | |  | |  | | |
| Question type | | | Array | | |  | |  | | |
| Validation | | | No | | |  | |  | | |

| Q22 | **Would you potentially use anti-CD20 therapy as a long-term strategy?** | | | | |  |
| --- | --- | --- | --- | --- | --- | --- |
|  | Yes | | | | Q22-1 |  |
|  | Yes, with restrictions | | | | Q22-2 |  |
|  | No | | | | Q22-3 |  |
|  | |  |  |  | | |
| Condition | | N/A |  |  | | |
| Question type | | Single Choice |  |  | | |
| Validation | | No |  |  | | |

| Q23 | **You have stated “Yes, with restrictions”.**  **Which of the following restrictions apply?**  Multiple responses possible | | | | |  |
| --- | --- | --- | --- | --- | --- | --- |
|  | Family planning | | | | Q23-1 |  |
|  | Safety | | | | Q23-2 |  |
|  | Lack of long-term data | | | | Q23-3 |  |
|  | Other (Open text) | | | | Q23-4 |  |
|  | |  |  |  | | |
| Condition | | IF Q22=2 |  |  | | |
| Question type | | Multiple Choice |  |  | | |
| Validation | | No |  |  | | |

| Q24 | **You have stated “No”.**  **Please state your reasons.**  Multiple responses possible | | | | |  |
| --- | --- | --- | --- | --- | --- | --- |
|  | Family planning | | | | Q24-1 |  |
|  | Safety | | | | Q24-2 |  |
|  | Lack of long-term data | | | | Q24-3 |  |
|  | Other (Open text) | | | | Q24-4 |  |
|  | |  |  |  | | |
| Condition | | IF Q22=3 |  |  | | |
| Question type | | Multiple Choice |  |  | | |
| Validation | | No |  |  | | |

| Q25 | **Would you in future discontinue ofatumumab therapy for any of the following reasons?**  Multiple responses possible | | | | |  |
| --- | --- | --- | --- | --- | --- | --- |
|  | Trying for a baby | | | | Q25-1 |  |
|  | Vaccination with inactivated vaccine scheduled | | | | Q25-2 |  |
|  | Vaccination with live vaccine scheduled | | | | Q25-3 |  |
|  | Surgery scheduled | | | | Q25-4 |  |
|  | Other | | | | Q25-5 |  |
|  | No (exclusive answer) | | | | Q25-6 |  |
|  | |  |  |  | | |
| Condition | | N/A |  |  | | |
| Question type | | Multiple Choice |  |  | | |
| Validation | | No |  |  | | |

| Q26 | **You have stated “Other”.**  **Please state your other reasons for discontinuing ofatumumab treatment.** | | | | |  |
| --- | --- | --- | --- | --- | --- | --- |
|  | Reason 1 | | | | Q26-1 |  |
|  | Reason 2 | | | | Q26-2 |  |
|  | Reason 3 | | | | Q26-3 |  |
|  | Reason 4 | | | | Q26-4 |  |
|  | Reason 5 | | | | Q26-5 |  |
|  | |  |  |  | | |
| Condition | | IF Q25=5 |  |  | | |
| Question type | | Multiple Open text |  |  | | |
| Validation | | Not mandatory |  |  | | |

| Q27 | **You have stated “Trying for a baby”.**  **For how long would you discontinue treatment?** | | | | |  |
| --- | --- | --- | --- | --- | --- | --- |
|  | Until after birth | | | | Q27-1 |  |
|  | Until the patient no longer breastfeeds | | | | Q27-2 |  |
|  | Until MS activity recurs | | | | Q27-3 |  |
|  | |  |  |  | | |
| Condition | | IF Q25=1 |  |  | | |
| Question type | | Single Choice |  |  | | |
| Validation | | No |  |  | | |

| Q28 | **You have stated the following reasons.**  **For how long would you discontinue treatment?**  (suffix = Monate) | | | | |  |
| --- | --- | --- | --- | --- | --- | --- |
|  | Vaccination with inactivated vaccine scheduled (IF Q25=2) | | | | Q2-1 |  |
|  | Vaccination with live vaccine scheduled (IF Q25=3) | | | | Q28-2 |  |
|  | Surgery scheduled (IF Q25=4) | | | | Q28-3 |  |
|  | Reason 1 (IF Q26=1) | | | | Q28-4 |  |
|  | Reason 2 (IF Q26=2) | | | | Q28-5 |  |
|  | Reason 3 (IF Q26=3) | | | | Q28-6 |  |
|  | Reason 4 (IF Q26=4) | | | | Q28-7 |  |
|  | Reason 5 (IF Q26=5) | | | | Q28-8 |  |
|  | |  |  |  | | |
| Condition | | IF Q25=2, 3, 4 or 5 |  |  | | |
| Question type | | Multiple Numeric Input |  |  | | |
| Validation | | No |  |  | | |

| Q29 | **How important do you think it is to determine the following parameters for monitoring the course of therapy in clinical practice?** | | | | | | | | |  |
| --- | --- | --- | --- | --- | --- | --- | --- | --- | --- | --- |
|  | **Very important** | **Important** | | **Neutral** | **Not all that important** | | **Not important** | | |  |
|  | Serum NfL | | | | | | | | Q29-1 |  |
|  | Total B-cell count | | | | | | | | Q29-2 |  |
|  | Number of B-cell-subtypes | | | | | | | | Q29-3 |  |
|  | | |  | | |  | |  | | |
| Condition | | | N/A | | |  | |  | | |
| Question type | | | Array | | |  | |  | | |
| Validation | | | No | | |  | |  | | |

| Q30 | **Before the injection of ofatumumab, would you in future use prior therapy with:**  Please only select the applicable options | | | | | | | |  |
| --- | --- | --- | --- | --- | --- | --- | --- | --- | --- |
|  | **First injection** | **Second injection** | | **Third injection** | | **From the fourth injection** | | |  |
|  | Steroids | | | | | | | Q30-1 |  |
|  | Antihistamines | | | | | | | Q30-2 |  |
|  | Paracetamol | | | | | | | Q30-3 |  |
|  | Other medicines | | | | | | | Q30-4 |  |
|  | | |  | |  | |  | | |
| Condition | | | N/A | |  | |  | | |
| Question type | | | Array (numbers) with checkbox | |  | |  | | |
| Validation | | | Not mandatory | |  | |  | | |

| Q31 | **Before the ofatumumab injections at home, would you advise patients to use prior therapy with:** | | | | | |  |
| --- | --- | --- | --- | --- | --- | --- | --- |
|  | **Yes** | | **No** | | | |  |
|  | Steroids | | | | | Q31-1 |  |
|  | Antihistamines | | | | | Q31-2 |  |
|  | Paracetamol | | | | | Q31-3 |  |
|  | Other medicines | | | | | Q31-4 |  |
|  | |  | |  |  | | |
| Condition | | N/A | |  |  | | |
| Question type | | Array | |  |  | | |
| Validation | | No | |  |  | | |

| Q32 | **How long would you in future monitor patients after their first ofatumumab injection in the surgery/hospital?**  Please give your response in the following format ”0:00“ (hours:minutes)  (suffix = Stunden:Minuten) | | | | |  |
| --- | --- | --- | --- | --- | --- | --- |
|  |  | | | | Q32-1 |  |
|  | |  |  |  | | |
| Condition | | N/A |  |  | | |
| Question type | | Numeric Entry |  |  | | |
| Validation | | Yes |  |  | | |

| Q33 | **Would you be in favour of self-injections at home after marketing authorisation of ofatumumab is granted?** | | | | |  |
| --- | --- | --- | --- | --- | --- | --- |
|  | No | | | | 33-1 |  |
|  | Yes, only after this many injections: (Open text) | | | | 33-2 |  |
|  | |  |  |  | | |
| Condition | | N/A |  |  | | |
| Question type | | Single Choice |  |  | | |
| Validation | | No |  |  | | |

| Q34 | **You have stated “No”.**  **For which reasons would you be against self-injections at home?**  Multiple responses possible | | | | |  |
| --- | --- | --- | --- | --- | --- | --- |
|  | Possibility of combining the injection with a monitoring visit | | | | Q34-1 |  |
|  | Lack of monitoring of correct therapy/injection time | | | | Q34-2 |  |
|  | Patient lacks cognitive or motor skills necessary for self-injection | | | | Q34-3 |  |
|  | Patient’s wishes | | | | Q34-4 |  |
|  | Other (Open text) | | | | Q34-5 |  |
|  | |  |  |  | | |
| Condition | | IF Q33=1 |  |  | | |
| Question type | | Multiple Choice |  |  | | |
| Validation | | No |  |  | | |

| Q35 | **In your opinion, would a reminder service for patients reminding them of the injections at home be beneficial?**  Multiple responses possible | | | | |  |
| --- | --- | --- | --- | --- | --- | --- |
|  | Yes, phone all | | | | Q35-1 |  |
|  | Yes, text reminder | | | | Q35-2 |  |
|  | Yes, smartphone app / push notification | | | | Q35-3 |  |
|  | Yes, email | | | | Q35-4 |  |
|  | No (Exclusive) | | | | Q35-5 |  |
|  | |  |  |  | | |
| Condition | | N/A |  |  | | |
| Question type | | Multiple Choice |  |  | | |
| Validation | | No |  |  | | |

| Q36 | **Did any of the following results of the ASCLEPIOS study positively surprise you?**  Multiple responses possible  Please click here for an overview of the results | | | | |  |
| --- | --- | --- | --- | --- | --- | --- |
|  | The low dose at which clinical relevance was achieved in the endpoints | | | | Q36-1 |  |
|  | The extent of the effects achieved on the endpoints | | | | Q36-2 |  |
|  | Not surprised by any result (Exclusive) | | | | Q36-3 |  |
|  | Other (Open text) | | | | Q36-4 |  |
|  | |  |  |  | | |
| Condition | | N/A |  |  | | |
| Question type | | Multiple Choice |  |  | | |
| Validation | | No |  |  | | |

| Q37 | **Please list the results of the study from “most convincing” (top) to “least convincing” (bottom).** | | | | |  |
| --- | --- | --- | --- | --- | --- | --- |
|  | Reduction in annual relapse rate | | | | Q37-1 |  |
|  | Reduction in lesions | | | | Q37-2 |  |
|  | Reduction in progression of disability | | | | Q37-3 |  |
|  | Safety | | | | Q37-4 |  |
|  | Other | | | | Q37-5 |  |
|  | |  |  |  | | |
| Condition | | N/A |  |  | | |
| Question type | | Ranking Question |  |  | | |
| Validation | | No |  |  | | |

| Q38 | **Which “other” results are you referring to in your list?**  If you are not referring to specific other results, you can skip this question. | | | | |  |
| --- | --- | --- | --- | --- | --- | --- |
|  |  | | | | Q38-1 |  |
|  |  | | | | Q38-2 |  |
|  |  | | | | Q38-3 |  |
|  | |  |  |  | | |
| Condition | | N/A |  |  | | |
| Question type | | Multiple Open text |  |  | | |
| Validation | | No |  |  | | |

Page 3

|  | **Please respond to the following questions in light of the SARS-CoV-2 pandemic.** |
| --- | --- |

| Q39 | **How do you handle distancing and hygiene rules on your premises during the SARS-CoV-2 pandemic?**  Multiple responses possible | | | | |
| --- | --- | --- | --- | --- | --- |
|  | The local premises have been reorganised | | | | Q39-1 |
|  | The local processes have been adapted, e.g. disinfection of treatment room surfaces after each patient | | | | Q39-2 |
|  | The staff works in groups in different shifts so that the groups cannot infect one another | | | | Q39-3 |
|  | Only a few patients are scheduled per day so that distancing rules can be complied with | | | | Q39-4 |
|  | No changes have been implemented (Exclusive) | | | | Q39-5 |
|  | Other (Open text) | | | | Q39-6 |
|  | |  |  |  | |
| Condition | | N/A |  |  | |
| Question type | | Multiple Choice |  |  | |
| Validation | | No |  |  | |

| Q40 | **You have stated “The local premises have been reorganised”.**  **Please specify this response.**  Multiple responses possible | | | | |
| --- | --- | --- | --- | --- | --- |
|  | There are multiple waiting rooms now | | | | Q40-1 |
|  | Patients must wait outside the surgery / hospital until they are called on | | | | Q40-2 |
|  | Fewer beds / couches than usual occupied in the room in which infusions are given | | | | Q40-3 |
|  | Other (Open text) | | | | Q40-4 |
|  | |  |  |  | |
| Condition | | IF Q39=1 |  |  | |
| Question type | | Multiple Choice |  |  | |
| Validation | | No |  |  | |

| Q41 | **Have you noticed changes to your patients’ behaviour during the SARS-CoV-2 pandemic?**  Multiple responses possible | | | | |
| --- | --- | --- | --- | --- | --- |
|  | Patients increasingly avoid monitoring visits, delay monitoring visits at the surgery/hospital or ask for telehealth visits | | | | Q41-1 |
|  | Patients avoid taking or being administered their MS therapy or want to delay it | | | | Q41-2 |
|  | Patients with acute MS symptoms/relapses avoid visits or want to delay visits | | | | Q41-3 |
|  | Patients have paused their MS therapy for a prolonged period of time or discontinued it | | | | Q41-4 |
|  | Patients increasingly ask for treatment alternatives to their current therapies | | | | Q41-5 |
|  | Patients increasingly require medical advice regarding their MS disease and the consequences of an immunomodulatory therapy | | | | Q41-6 |
|  | No (Exclusive) | | | | Q42-7 |
|  | Other (Open text) | | | | Q42-8 |
|  | |  |  |  | |
| Condition | | N/A |  |  | |
| Question type | | Multiple Choice |  |  | |
| Validation | | No |  |  | |

| Q42 | **Have you made changes to the treatment of MS patients (in consultation with the patient) during the SARS-CoV-2 pandemic?** | | | | | | | |
| --- | --- | --- | --- | --- | --- | --- | --- | --- |
|  | **No** | **In isolated cases** | | **Frequently** | | **In the majority of cases** | | |
|  | Monitoring visits are postponed | | | | | | | Q42-1 |
|  | Monitoring visits are performed as telehealth visits | | | | | | | Q42-2 |
|  | MS infusion appointments are postponed | | | | | | | Q42-3 |
|  | Alternative therapies which the patient can also take/use at home are initiated | | | | | | | Q42-4 |
|  | Other (Open text) | | | | | | | Q42-5 |
|  | | |  | |  | |  | |
| Condition | | | N/A | |  | |  | |
| Question type | | | Array | |  | |  | |
| Validation | | | No | |  | |  | |

| Q42B | **Which “other” changes are you referring to?**  **If you are not referring to specific other changes, you can skip this question.** | | | | |
| --- | --- | --- | --- | --- | --- |
|  | Change 1: | | | | Q42-1 |
|  | Change 2: | | | | Q42-2 |
|  | Change 3: | | | | Q42-3 |
|  | |  |  |  | |
| Condition | | N/A |  |  | |
| Question type | | Multiple Open text |  |  | |
| Validation | | No |  |  | |

| Q43 | **Do you have any concerns regarding using immunomodulatory MS medicines during the SARS-CoV-2 pandemic?** | | | | | | | | |
| --- | --- | --- | --- | --- | --- | --- | --- | --- | --- |
|  | **No** | **In general** | | **When re-titrating therapy** | **In patients belonging to the SARS-CoV-2 risk group (e.g. pre-existing disorders, elderly, etc.)** | | **In case of particularly exposed patients (e.g. nurses, cashiers etc.)** | | |
|  | Interferon ß, glatiramer acetate | | | | | | | | Q43-1 |
|  | Fumaric acid, teriflunomide, fingolimod, siponimod | | | | | | | | Q43-2 |
|  | Cladribine, natalizumab, alemtuzumab, ocrelizumab | | | | | | | | Q43-3 |
|  | | |  | | |  | |  | |
| Condition | | | N/A | | |  | |  | |
| Question type | | | Array | | |  | |  | |
| Validation | | | No | | |  | |  | |

| Q44 | **Does a longer stay on the premises of a hospital / surgery such as e.g. during a DMT infusion or a clinical study visit constitute a risk in your opinion?** | | | | |
| --- | --- | --- | --- | --- | --- |
|  | Yes | | | | Q44-1 |
|  | No | | | | Q44-2 |
|  | |  |  |  | |
| Condition | | N/A |  |  | |
| Question type | | Single Choice |  |  | |
| Validation | | No |  |  | |

| Q45 | **In light of the SARS-CoV-2 pandemic, do you see the possibility of an MS therapy that can be self-injected / taken at home as an advantage over MS therapies administered as infusions?** | | | | |
| --- | --- | --- | --- | --- | --- |
|  | Yes | | | | Q45-1 |
|  | No | | | | Q45-2 |
|  | |  |  |  | |
| Condition | | N/A |  |  | |
| Question type | | Single Choice |  |  | |
| Validation | | No |  |  | |

| Q46 | **Do you assume that the SARS-CoV-2 pandemic will have a long-term impact (beyond the duration of the SARS-CoV-2 pandemic) on your view of therapies that can be used at home?** | | | | |
| --- | --- | --- | --- | --- | --- |
|  | Yes | | | | Q46-1 |
|  | No | | | | Q46-2 |
|  | |  |  |  | |
| Condition | | N/A |  |  | |
| Question type | | Single Choice |  |  | |
| Validation | | No |  |  | |

| Q47 | **Have you made changes to your clinical study activities during the SARS-CoV-2 pandemic?**  Multiple responses possible | | | | |
| --- | --- | --- | --- | --- | --- |
|  | Clinical study visits are postponed | | | | Q47-1 |
|  | Clinical study visits are performed as telehealth visits | | | | Q47-2 |
|  | Clinical study visits are performed as home visits | | | | Q47-3 |
|  | Clinical study subjects are given the medication as part of a home visit | | | | Q47-4 |
|  | The medication is delivered to clinical study subjects’ homes | | | | Q47-5 |
|  | Recruitment has been stopped | | | | Q47-6 |
|  | Clinical study has been discontinued | | | | Q47-7 |
|  | No further participation in new clinical studies for the time being | | | | Q47-8 |
|  | No (Exclusive) | | | | Q47-9 |
|  | Other (Open text) | | | | Q47-10 |
|  | |  |  |  | |
| Condition | | N/A |  |  | |
| Question type | | Multiple Choice |  |  | |
| Validation | | No |  |  | |
